# Automatic heart rate clamp: A practical tool to control internal and external training loads during aerobic exercise

**DOI:** 10.3389/fphys.2023.1170105

**Published:** 2023-04-07

**Authors:** Siu Nam Li, Peter Peeling, Brendan R. Scott, Jeremiah J. Peiffer, Alex Shaykevich, Olivier Girard

**Affiliations:** ^1^ School of Human Sciences (Exercise and Sports Science), The University of Western Australia, Perth, WA, Australia; ^2^ Department of Sport Science, Western Australian Institute of Sport, Mount Claremont, WA, Australia; ^3^ Murdoch Applied Sport Science Laboratory, Discipline of Exercise Science, Murdoch University, Perth, WA, Australia; ^4^ Centre for Healthy Ageing, Murdoch University, Perth, WA, Australia; ^5^ Perron Institute for Neurological and Translational Science, Perth, WA, Australia

**Keywords:** training loads, clamped heart rate, environmental stress, hypoxia, exercise prescription

## Background

Prescribing and monitoring exercise intensity is a fundamental competency for exercise physiologists and sport scientists working across a range of populations to achieve desired clinical and/or performance outcomes (Impellizzeri et al., 2019). A variety of external and internal load metrics can be considered for this purpose. External load refers to the objective measures of the work performed (i.e., sustained mechanical output), whereas internal load represents the physiological and perceptual responses to that external load (Foster et al., 2017). Said differently, external load answers the question “*How was training implemented?*” (i.e., what happens on the ‘outside’), while internal loads determine “*Did the individual respond to the training as planned?*” (i.e., what happens on the ‘inside’). Although internal load during exercise relates closely to the psycho-physiological adaptations elicited (Impellizzeri et al., 2019), exercise intensity is often controlled from external load metrics. To sustain cycling exercise intensity at lactate threshold, power output (PO) corresponding to lactate threshold is commonly used as a surrogate measure, resulting in external load as a proxy to elicit a given internal load. However, when exercise intensity is prescribed from an external load metric, a dissociation may occur between external and internal loads. For instance, cardiovascular drift can manifest 10 min after the onset of moderate-intensity exercise, which reflects a progressive increase in internal load for a constant external load (Ekelund, 1967). Internal load could therefore be disproportionally increased beyond the required stimulus (i.e., above threshold), such that an expected physiological adaptation may not be elicited. As a result, internal load metrics are more commonly used to prescribe exercise stimulus in order to achieve the desired training outcomes. This article will explore the use of alternative strategies to prescribe and monitor exercise intensity during aerobic exercise.

### Internal load metrics

In order to carefully control internal load during an aerobic exercise bout, it is possible to continually adjust external load so that a pre-planned internal load is reached and maintained. For example, in a laboratory setting, external load can be altered by increasing or decreasing belt speed on a treadmill or resistance on a cycle-ergometer. A variety of physiological variables (i.e., heart rate, blood lactate concentration, muscle oxygenation and activation, and oxygen consumption) can be considered to reflect internal load. Any selected variable would then need to be measured relatively easily and continuously, and be relevant to the sporting context, for their adoption by a large number of end-users in real-world training scenarios. For example, although blood lactate can be measured continuously (Cass and Sharma, 2017), its response time to changes in exercise intensity is relatively long compared to other physiological measures (Bentley et al., 2007). While oxygen consumption has a quick response time to changes in exercise intensity, collecting gas exchange requires an expensive metabolic cart with participants also constrained to wear a face mask that likely restricted fluid intake during exercise. Alternatively, HR is an easy-to-measure, non-invasive, and inexpensive physiological variable to represent internal load. By wearing a chest or watch strap monitor, continuous HR measurements can be readily obtained to inform exercise prescription for a wide range of active populations (sedentary to elite athletes). Of note, the simplicity of this measure also enables simultaneously tracking a large number of individuals training together. Therefore, for most exercise bouts performed on an ergometer, HR is considered a suitable indicator of internal load to monitor and control aerobic exercise intensity.

### Current practices to control heart rate during exercise

Controlling for internal load derived from HR metrics is achieved *via* manual adjustment of external load. To guide training delivery, HR zones defined according to noticeable physiological landmarks, such as ventilatory thresholds (VT), are often considered. By using HR zones relative to VT (i.e., 10–15 bpm below VT1, 10–20 bpm above VT1, and below VT2, 10–15 bpm above VT2), improvements in maximal oxygen uptake following a 12-week protocol have been reported (Wolpern et al., 2015). To successfully maintain HR within the prescribed zones, investigators had to regularly adjust treadmill speed and gradient. However, the actual HR response during sessions is unknown because intra-session HR data was not reported. Therefore, the accuracy of manual adjustment to maintain internal load is undetermined. A potential limitation of manual adjustment of external workload is that HR is prescribed in zones, while this approach may not be valid when the maintenance of HR needs to be more accurate (i.e., a specific HR value). For example, given large differences in inter-individual responses to standardized exercise training (prescribing HR in zones), the approach of prescribing exercise intensity must be tailored and carefully controlled to achieve the desired outcomes (Mann et al., 2014). Consequently, the inter-individual variability of exercise responses may be reduced when HR is controlled to a fixed value when compared to HR controlled to zones. Another limitation of this method is that the adjustment to external load relies on the discretion of the investigators, which may introduce inter-rater reliability issues. For example, different experimenters may adjust external workload at different magnitudes to maintain the target HR (i.e., increasing/decreasing treadmill speed by 0.1 versus 0.5 km h^−1^ or resistance by 1 versus 5 W). Other drawbacks of manual adjustment include the frequency of when external workloads are modified and the HR averaging period. For instance, Racinais et al. (2021) altered cycling PO every 30 s to maintain a HR corresponding to 60% of maximal oxygen uptake during a study requiring fixed HR across various environmental conditions. Because HR may have changed with each 30-s epoch, the fluctuations in HR may be higher than if HR was adjusted continuously. As such continuous and immediate adjustments of external workload are needed if HR is to be precisely controlled.

## Automatic heart rate clamp

A possible solution to solve some of the aforementioned issues associated with controlling HR is the use of an automatic HR clamp. When using this method, after a target HR is first set, a computer program would instantaneously adjust external workload (e.g., cycling PO) so that participants may reach and maintain this pre-planned HR response. An initial attempt to achieve this was made by Kawada et al. (1999), who used a servo-controller to clamp HR in healthy individuals cycling an ergometer. In this study, the time taken to reach 90% of target HR corresponding to 60% and 75% HR_max_ was ∼136 s, and the difference between target and actual HR was ∼3 bpm. These findings suggest that an automatic HR clamp likely allows HR to be precisely regulated. An advantage of this approach is also that the difference between target and actual HR is calculated almost instantaneously at each timepoint (0.1 Hz), so that reactive adjustments to cycling PO can be implemented immediately. Additionally, the control of HR dictated by an algorithm removes any inter-rater reliability issues. When exercise is prescribed at a constant sub-maximal exercise intensity, an automatic HR clamp likely represents a suitable method to tightly regulate internal load.

In addition to continuous exercise, the HR clamp approach has successfully been implemented to control intermittent exercise intensity (i.e., interval training sessions). For instance, through the use of an automatic HR clamp tool Hunt and Hurni (2019) had participants perform four intervals alternating between active (∼140 bpm) and passive (∼120 bpm) phases. The reported mean tracking error of ∼3.1 bpm would suggest that HR can be closely controlled even when the target HR fluctuates during exercise. Overall, an automatic HR clamp approach can be successfully implemented for different modes of aerobic training.

### Comparing automatic and manual control of power output to reach a pre-planned HR

**FIGURE 1:**
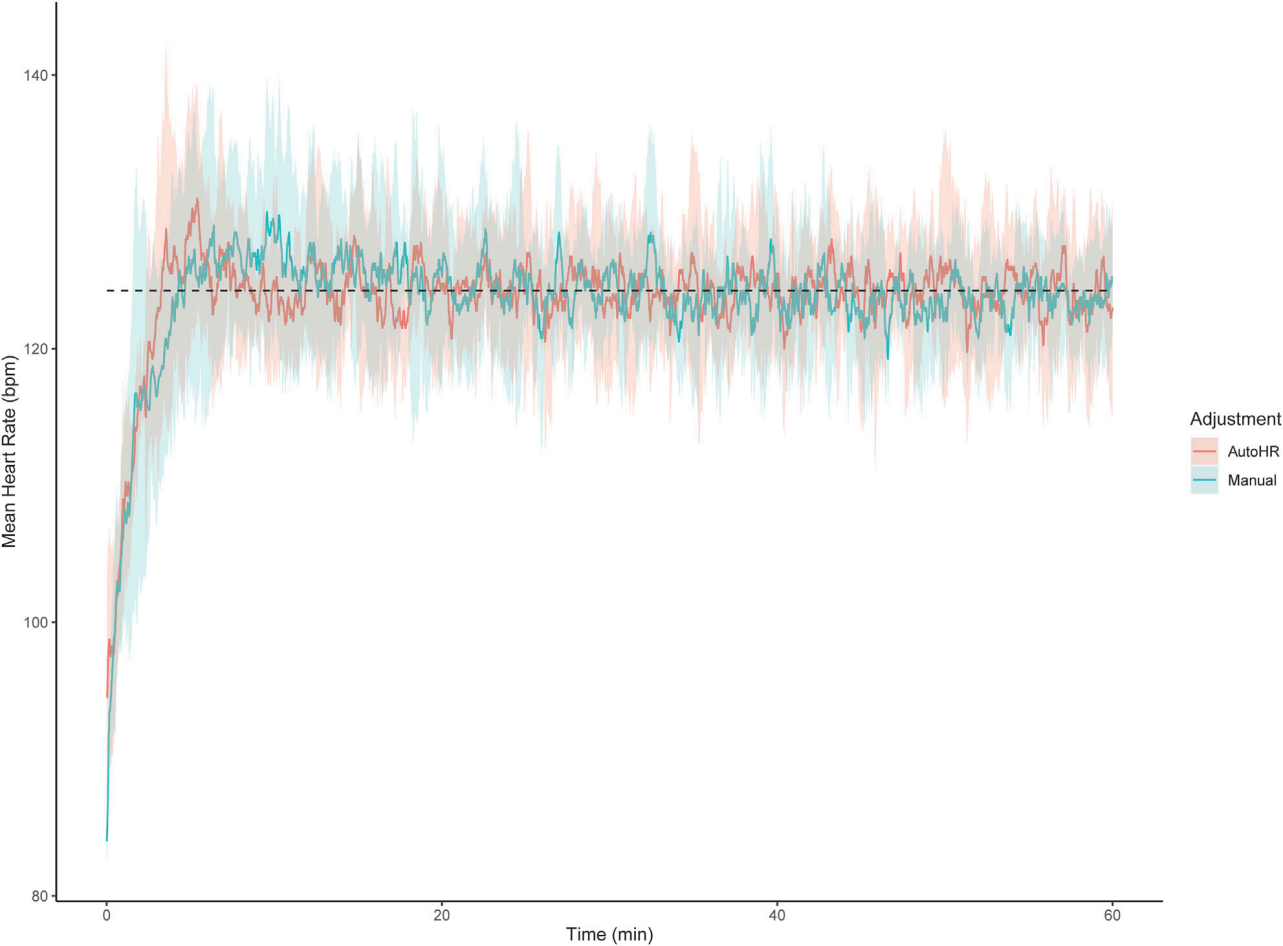
Pilot data depicting average heart rate across 60 min of heart rate clamped cycling between automatic (AutoHR) and manual control of power output. Dashed line represents average target heart rate (124 bpm). The shaded areas represent the standard deviation of mean actual heart rate.

Previously, direct comparisons have not been made between manual and automatic control methods of external workload to maintain a target HR. Here, we present pilot data from five participants comparing an automatic and manual control of PO to reach a pre-planned HR (Figure 1). We specifically compared an automatic HR clamp (AutoHR) installed on an iPod Touch (Apple Incs., Cupertino, CA, United States) against manual adjustments to external workload by the same experimenter during 60 min of cycling at individual HR corresponding to 80% VT (∼40% peak oxygen consumption) in trained males. The AutoHR app uses a standard Bluetooth low energy profile to control the resistance of the ergometer, which is supported by many off-the-shelf cycling ergometers (e.g., Wahoo kickR, Tacx Neo). To maintain HR, the AutoHR application calculates the HR error (difference between current and target HR), computes target wattage, and sets the ergometer’s resistance accordingly at 5-s intervals by implementing a proportional-integral controlled based on the principles of Kawada et al. (1999). Furthermore, the proportional integral controller is defined by the equation: *u(t) = Kpe(t) + Ki*∫*e(t)dt*, where *Kp* and *Ki* are the proportional and integral gains, respectively; *e(t)* is the error at time *t* defined as the difference between the measured (HR from the chest strap monitor) and target (target HR set on the app) values of the observed variable at time *t*, and ∫*e(t)d* is the integral of the error term with respect to time (Aström and Hägglund, 2001). From the equation, the app calculates the adjustments in ergometer resistance required to match measured and target HR. The increase in PO during the first 3 min of exercise from a resting state with this HR clamp procedure was strictly replicated for manual adjustment trials. Interestingly, after the initial 3 min, root mean square error for the automatic HR clamp and manual adjustment methods were ∼2 ± 2.0 and ∼4 ± 2.0 bpm, respectively. These observations suggest that the AutoHR clamp method allows a more precise control of the HR response compared to manual adjustment of external load. However, as the study was a pilot test with only five participants, further investigations are required to confirm these results.

## Practical applications

For a given external workload, many factors can affect internal load and subsequently the training outcomes. For instance, individuals’ training status, psychological status, health, nutrition, environment, and genetics can all affect the internal load response to training (Impellizzeri et al., 2019). Consequently, suggested practical applications of the automatic HR clamp include automatically controlling the internal load of exercise across various environmental conditions during both acute and chronic interventions.

### Acute interventions

Environmental stressors (i.e., cold, hot, hypoxia, pollution) can affect the relationship between internal and external loads during exercise (Impellizzeri et al., 2019). When compared to ambient near sea-level conditions, exposure to either hot or hypoxic environments induce a disproportionate elevation of the internal load response to exercise for a given external load in a dose-response manner (Buchheit et al., 2013). In this context, a HR clamp approach may be useful to monitor and reduce the external workload in response to these challenging environments, eliciting a comparable cardiometabolic response in reference to controls (Girard et al., 2021). Therefore, by using a HR clamp, the desired physiological responses from exercise can be achieved, regardless of the severity of the environmental stress.

### Chronic interventions

By taking advantage of the *higher-than-normal* stress of exercising in challenging environmental conditions, a HR clamp approach may allow similar (or eventually better) post-intervention physiological and/or functional benefits, despite a lower overall mechanical stress during actual training sessions (Girard et al., 2021). To date, there is no published evidence of the chronic effects of exercise that uses a HR clamp across different environmental conditions. Nonetheless, similar maximal oxygen uptake improvements were achieved in overweight-to-obese individuals after training at HR corresponding to 65% maximal oxygen uptake (albeit *via* manual adjustment of external load) between sea-level and moderate hypoxia (Wiesner et al., 2010). Remarkably, the hypoxia group had greater body composition improvements (i.e., % fat free mass, waist circumference) when compared to the sea-level group despite a 17.5% reduction in external load. An automatic HR clamp approach may therefore become advantageous to more carefully control HR to maintain internal load across a range of environmental conditions during chronic interventions.

In clinical populations (e.g., cardiometabolic pathologies, pulmonary, and cardiac rehabilitation), the automatic HR clamp can be very useful for a strict control of exercise intensity, which is pertinent given the risk associated with exceeding prescribed intensities in such populations (Warburton et al., 2011). Additionally, the algorithm within the AutoHR app may be further improved by taking into account any known chronic abnormalities in HR during exercise and adjust external load accordingly. Therefore, the automatic HR clamp can help to tightly control exercise intensity in clinical populations.

Logistically, the automatic HR clamp is relatively easy to implement during group training sessions compared to manual adjustment to external load. For example, for a group of participants exercising simultaneously, no additional practitioners would need to be present to adjust external load for each individual when the automatic HR clamp is used. Comparatively, manual adjustment likely requires one practitioner for each person, which may not be feasible (or even possible) for large groups. One advantage of the automatic HR clamp is chronic interventions is therefore to control exercise intensities in large cohorts training together.

## Conclusion

We argue that internal load of exercise can be easily controlled through the adjustment of external workload *via* automatic HR clamps. Such method allows for relative exercise intensity to be maintain constant both within and between exercise sessions, notably when training with additional environmental stress. Moving forward, it is our hope that automatic HR clamp approaches will be more commonly considered as a means of prescribing exercise intensities in research and practical settings when ergometer-based exercises are performed.
